# What makes clinical machine learning fair? A practical ethics framework

**DOI:** 10.1371/journal.pdig.0000728

**Published:** 2025-03-18

**Authors:** Marine Hoche, Olga Mineeva, Gunnar Rätsch, Effy Vayena, Alessandro Blasimme

**Affiliations:** 1 Department of Computer Science. Biomedical Informatics Group, ETH Zurich, Zurich, Switzerland,; 2 AI Center, ETH Zurich, Zurich, Switzerland,; 3 Department of Health Sciences and Technology. Health Ethics and Policy Lab, ETH Zurich, Zurich, Switzerland; Mayo Clinic Rochester: Mayo Clinic Minnesota, UNITED STATES OF AMERICA

## Abstract

Machine learning (ML) can offer a tremendous contribution to medicine by streamlining decision-making, reducing mistakes, improving clinical accuracy and ensuring better patient outcomes. The prospects of a widespread and rapid integration of machine learning in clinical workflow have attracted considerable attention including due to complex ethical implications–algorithmic bias being among the most frequently discussed ML models. Here we introduce and discuss a practical ethics framework inductively-generated via normative analysis of the practical challenges in developing an actual clinical ML model (see case study). The framework is usable to identify, measure and address bias in clinical machine learning models, thus improving fairness as to both model performance and health outcomes. We detail a proportionate approach to ML bias by defining the demands of fair ML in light of what is ethically justifiable and, at the same time, technically feasible in light of inevitable trade-offs. Our framework enables ethically robust and transparent decision-making both in the design and the context-dependent aspects of ML bias mitigation, thus improving accountability for both developers and clinical users.

## Author summary

Artificial intelligence (AI) can be fruitfully integrated into clinical workflow, improving efficiency and accuracy in clinical decision-making. However, AI systems–such as machine learning algorithms–come with very well know limitations. One of the most discussed limitations of machine learning, in medicine and beyond, is the documented tendency of such systems to exhibit bias in their performance, as well as in their practical outcomes. Unfair medical algorithms can reproduce or exacerbate existing health inequalities by causing AI to perform differently for different patient groups, or resulting in unfair health outcomes. This paper offers a framework to ethically assess and minimize AI bias in clinical practice. Our framework integrates ethical and technical requirements, thus representing a practical contribution to ensure fairness in clinical AI.

## Introduction: Fairness in clinical ML models

Fairness is often discussed as a specific requirement of justice in matters of resource distribution or allocation, as well as with respect to the differences in resources or goods between individual and population groups. Justice refers in general to giving people what is due to them, such as goods they are entitled to, or punishments they deserve [1]. Fairness specifies that certain individual or group characteristics (such as gender, sexual orientation, age, religion, ethnic origin and the like) should not influence how one is treated with respect to what is due to them or what they are entitled to–that is, that people should not have or attain less or more in virtue of those characteristics.

In the context of clinical machine learning (ML), fairness means ensuring that a model performs similarly for different types of patients and that characteristics such as gender, sexual orientation, age, religion, ethnic origin and the like do not skew a model’s output, nor the outcomes of its use. Differences in model performance or in model outcomes across any subgroup of patients should be considered from an ethical point of view [2]–even if they do not affect socially disadvantaged groups, and even if–contrary to what others have argued (3,4)–they go to the advantage of patients belonging to unprivileged groups.

However, not all differences in model performance have equal ethical weight [3,4] especially when mitigation measures would result in deteriorated performance, thus giving rise to trade-offs. Moreover, some differences among different subgroups may be due to underlying clinical or biological characteristics, and may not be avoidable. For instance, predicting clinical outcomes may be harder for certain classes of patients than others. Those kinds of biases are due to the nature of the underlying phenomenon and while not unfair [5], they should still be documented for optimized clinical use–for instance by restricting model implementation.

*Model fairness* (often also called group fairness) refers to ensuring that ML models have similar performance for different patient groups. *Outcome fairness*, instead, means controlling for disparities in health outcomes in different patient groups for which a model is used. Different formalizations of fairness are defined in Table 1. While interconnected, model and outcome fairness are two separate problems [6]. Mitigating model performance bias is likely to result in more equitable health outcomes. However, additional factors may have to be considered. Moreover, fair outcomes do not necessarily signal that model performance is indeed also fair, as they may be due to implementation features that compensate for model unfairness.

**Table 1:** Different mathematical formalization of model fairness. TPR: True Positive Rate; FPR: False Positive Rate; FNR: False Negative Rate; TNR: True Negative Rate; PPV: Positive Predictive Value; NPV: Negative Predictive Value; AUROC: area under the receiver operating characteristic (ROC) curve (as a function of TPR and FPR); AUPRC: area under the precision-recall curve (as a function of PPV and TPR).

Fairness metrics	Definition
*Equalized odds*	same AUROC
*Equal opportunity*	Same TPR (recall or sensitivity) = 1–FNR
*Precision-recall parity*	same AUPRC
*Predictive equality*	Same FPR (fall-out, probability of false alarm) = 1–TNR
*Predictive parity*	Same PPV (precision)
*Equal conditional use accuracy*	Same PPV (precision) AND NPV
*Equal selectivity*	Same TNR (specificity) = 1–FPR

## Methods

This paper provides a conceptual framework that can be generally employed in clinical practice irrespective of medical subfield. A conceptual framework does not consist of a detailed mathematical pipeline, nor it includes specific debiasing techniques. Rather, a conceptual framework is used to decide between different possible mathematical formalization of fairness and debiasing techniques. Our framework was built inductively based on a normative analysis of the practical challenges posed by devising and calibrating the model described in the case study (see Boxes). Our normative analysis is based on philosophical accounts of structural injustice [7] and justice in healthcare [8,9]. We have followed a deontological normative approach [10,11], based on conceptual analysis techniques developed in the field of principle-based bioethics [1,12,13]. More specifically, we did not deduce our framework from our use case. Rather, based on theoretical assumptions about structural injustice, we inductively extracted salient normative features from the use case and abstracted them to formulate normative principles to be applied beyond the use case itself. As a result, none of the action-guides we propose is specific to the field of the use case (i.e., intensive care medicine).

In the paper, we employ the use case merely to illustrate the framework with concrete examples of how bias can be measured across different mathematical formalization and different patient characteristics. Full methodological details about the case study are provided in the boxes.

In what follows we illustrate how potential sources of bias can be identified (Section 1), measured and mitigated when bias depends on model features (Sections 2 and 3), as well as when it depends on the conditions of its implementation and use (Section 4). We identify action points that specify the requirements of fairness for each step of our framework, and we show how to use it through a case study based on a ML model to predict circulatory failure in patients in intensive care units (see Box 1).

**Box 1. Case study description**

A ML model provides early warning of imminent (12 hours) circulatory failure in patients admitted to an intensive care unit (ICU) for any clinical reasons [14]. We obtained anonymized high-resolution times-series data (HiRID) [15] with monitoring, lab measurements, medications, observations as well as static clinical attributes (admission reason, admission type - emergency or elective - surgical status - i.e., whether the patient had underwent surgery) and demographics (age, gender) from Inselspital in Bern (Switzerland) for model training and validation. Data were collected between January 2008 and June 2016. This dataset is publicly available for research use.

Our training dataset does not contain ground-truth labels for circulatory failure. We thus used a clinician-approved proxy, namely arterial lactate level above 2 mmol/L (irregular sampling upon doctor’s request) and mean arterial pressure below 65 mmHg (sampling rate: 2 minutes). The model outputs a prediction every 5 minutes. If the prediction is above the threshold the model triggers a warning; if the prediction is below the threshold, the model does not provide any feedback.

## Results

### 1. Identifying sources of bias

Identifying possible sources of bias in training datasets is a first step for the construction of a fair clinical ML model [16,17].

=> **[A1] Developers should identify potential sources of bias to define for which classes of patients the model may under-perform.**

Most ethical discussions about ML bias focus on insufficient socio-demographic representativeness in training data as the principal cause of ML bias. This problem affects in particular minorities and socially disadvantaged groups that, typically, are underrepresented in available datasets (minority bias, Table 2). Training ML models on more balanced datasets is more likely to yield models that perform evenly across different socio-demographic groups. More inclusive training datasets, however, tend to also include protected attributes (such as age, gender, sex and sexual orientation, ethnicity, race, religion, etc.). Some argue that protected characteristics should not be taken into account in the training process, so as to avoid models that unfairly associate those features to their output [16]. However, obfuscating protected attributes such as age or gender in medical ML may deteriorate model performance in ways that are also ethically problematic. And, while it is possible that more inclusive datasets could make ML models fairer, sometimes available datasets are simply not equally representative of all demographics. In such cases, under-represented groups should be identified, so as to enable further testing of model performance and outcomes across different patient cohorts.

**Table 2:** Potential sources of bias affecting model fairness (section 3) and outcome fairness (section 4).

Type of bias	Definition	Ethical challenges
*Affecting model fairness (* ***section 3*** *)*		
***Minority bias***	Some categories of the population are insufficiently represented in training and validation datasets	Justice: the model may underperform for non-represented populations, such as minorities or socially disadvantaged groups, which could result in exacerbating existing health inequalities.
***Protected attributes bias***	Protected attributes (such as gender or ethnicity) determine model output	Justice: protected attributes may give rise to models that discriminate against specific groups. Non-maleficence: removing protected attributes that may have medical relevance deteriorates model performance.
***Missing data bias***	The absence of certain data points in a dataset due to nonrandom factors like patient non-compliance, loss to follow-up, inconsistent data collection practices, data entry errors specific to a given patient group, etc.	Non-maleficence: missingness deteriorates model performance for specific patient groups. Justice: missingness can affect already disadvantaged populations more than others thus perpetuating patterns of structural injustice.
***Label bias***	A clinical label or a clinical label proxy can work well for certain patients but not for others.	Justice: if the choice of label proxies does not capture the true label well for all patients, then the model will not be equally accurate for all patients.
*Affecting outcome fairness (* ***section 4*** *)*		
***Training-serving skew***	The patient population on which the model is used differs from the population on which the model has been developed	Justice: the model may fail to yield similar health outcomes for all patient groups. Non-maleficence: the model may lead to worse health outcomes for underrepresented groups of patients.
***Automation bias***	Model users over-rely on the model predictions	Justice and non-maleficence: patients for which the model is less accurate will have worse health outcomes.
***Dismissal bias***	Model users, consciously or unconsciously, fail to attend to the model’s predictions or decisions	Justice and non-maleficence: more at-risk patients can be harmed and experience worse health outcomes.

It is also possible that training and validation datasets do not contain sufficient socio-demographic information, to enable developers to control for minority bias. In such cases the effects of minority bias should be monitored in terms of health outcomes when the model is in use (section 4).

Beyond minority bias, other well-documented sources of data-related bias include protected attribute bias, missing data bias and label bias [18] (Table 2 for definitions). Developers should carefully consider whether such forms of bias may affect their training datasets so as to determine for which patient classes their model may underperform (Box 2).

The identification of a particular source of bias in training and validation data is not a sufficient reason to foreclose the use of such data for model development. The fundamentally opaque nature of ML models makes it particularly hard to predict, by simply looking at training data composition, if and how ML bias will manifest in a model. Moreover, some variation in model performance may reflect natural characteristics of the observed phenomenon rather than under- or over-sampling.

Identifying possible sources of bias in training and validation data serves the purpose of defining which classes of patients or which patient attributes should be used to measure biased model performance (section 2). It should be noted that specific patterns of disadvantage often result from the intersection of different characteristics. According to intersectional accounts of justice certain individuals are particularly vulnerable to patterns of systematic disadvantage due to the multiple, overlapping forms of inequality and discrimination that they face. Therefore, it is important to identify (and tests) not only individual attributes (e.g., age, gender, ethnicity), but also how their combination further amplifies disadvantage.

Once potential sources of bias have been identified, the second step to minimize unfairness is to measure how bias manifests itself in terms of different performance for different classes of patients (section 3). Potential sources of bias linked to model use such as, training-serving skew, automation bias and dismissal bias (Table 2) can instead best be addressed upon model implementation [18] (section 4).

**Box 2. Bias identification**

Variables such as ethnicity and socio-economic status were not reported in our training dataset and could thus not be controlled for. This model may thus suffer from minority bias, which needs to be taken into account upon implementation.

The sex distribution in our data is 65% male and 35% female patients. We thus need to control for protected feature bias between male and female patients

The age of patients in our data is quite diverse. We therefore have to control for protected feature bias among different age groups.

Since we use a clinical proxy, we checked for potential label bias in our proxy definition and found out that for neurological patients–who have increased arterial pressure due to medications used to keep their circulatory pressure higher for clinical reasons–the model may produce inaccurate outcomes. We thus need to control for label bias across different clinical indications (reasons for admission).

### 2. Model fairness: Measuring model fairness

To measure if a model is actually biased against any patient class, it is necessary to adopt a metric to compare model performance across relevant patient classes. Classes such as gender or ethnicity should routinely be tested whenever possible as they are known to account for substantial bias in medicine. Available formalizations of algorithmic fairness include: equalized odds, equal opportunity, precision-recall parity, predictive equality, predictive parity, equal conditional use accuracy, and equal selectivity (Table 1 for definitions) [19]. From a mathematical point of view, it is impossible to satisfy all the above-mentioned formalizations at the same time [20–23]. As a consequence, some degree of disparity in the way a model performs for different groups under different formalizations of fairness is unavoidable. Therefore,

=> **[A2.1] Developers should define which mathematical formalization of fairness is best suited to the clinical task at hand.**

This entails that some ethical trade-off will most likely have to be accepted. General criteria to determine the best possible fairness metric are not available. Clinical and ethical considerations can guide the choice of the most appropriate measure of model performance in specific cases [24]. For instance, for a model that detects a serious condition requiring immediate intervention, a heightened fall-out (probability of false positive or false alarm) measured as predictive equality is more acceptable than a higher miss-out (probability of false negative).

Furthermore, one needs to consider whether the condition the model is being used for is more prevalent in certain patient classes over others. If so, it is preferable to choose formalizations of fairness that are not affected by the difference in prevalence (e.g., equal opportunity) or to normalize the chosen metric for prevalence.

=>**[A2.2] Developers should comparatively assess model performance across identified patients classes based on the fairness metric(s) they have selected.**

For each defined patient class, it is possible to compute the metric values for class members versus the rest of the cohorts. Performance disparities can be quantified by


$$\Delta =\left|\text{median}{\left\{{\text{metric}}_{p\in S_{n}\cap G}\right\}}_{n=1}^{N}-\text{median}{\left\{{\text{metric}}_{p\in S_{n}\cap \bar{G}}\right\}}_{n=1}^{N}\right|,$$

Where *p* is a patient or data point, $S_{n}$ is the *n* -th bootstrapped sample, *N* is the total number of bootstrapped samples, *G* is the studied patient class, $\bar{G}$ is the rest of the population. This formula quantifies disparities by comparing median metric values between one class and the rest of the population; an alternative is comparing to the entire population.

While we focus on performance deltas that are directly interpretable and allow calculation of the number of patients affected, we note that standardized effect sizes being unitless, it enables comparisons across different metrics and studies. Therefore, we consider both approaches complementary and suggest that standardized effect sizes can be reported alongside performance deltas for a more comprehensive understanding.

Note that multiple metrics can be measured to observe how the model behaves in general, to check if bias manifests for any given group across different formalizations of fairness. Such a comparative approach may help in the selection of the most effective debiasing technique (see below, Section 3). To support the practical implementation of our framework, tools such as FAMEWS [25] can facilitate quantitative analyses of fairness-performance trade-offs in clinical machine learning models.

### 3. Improving model fairness

Not all disparities are equally relevant from an ethical point of view, and some biases may be caused by clinical or biological characteristics, such as the prevalence of a condition in a given subgroup or genetic factors. In such cases, differences are not unfair and require caution in model implementation rather than bias mitigation per se. Developers can consult with clinical experts to clarify when this may be the case. In all other cases, since strict equality is not possible, choices have to be made. Ethical criteria should determine whether and to which extent measured biases should be addressed.

=> **[A3.1] Developers should decide which of the identified disparities across patient groups should be corrected.**

We refrain from introducing absolute numerical thresholds as a way to identify disparities that warrant ethical considerations. From an ethical point of view, a *statistically significant* difference in the way an algorithm performs for different patient groups generally indicates that some bias mitigation may be appropriate. Developers, however, should also assess the *magnitude* of the difference. Minor differences need not be corrected, even if they are statistically significant, because they are unlikely to cause tangible disadvantage. Also the *impact* of the bias matters, that is, how large is the group of people that the model discriminates against and how big are the risks resulting from the behavior of the model. If in a specific clinical setting the model will encounter only a very limited number of patients belonging to the group for which the model underperforms, correcting for that bias may have more negative than positive consequences in the aggregate if it results in overall worse accuracy. In that case, the risk of biased performance should at any rate be documented so as to restrict model use. Conversely, if the model is inaccurate for a larger cohorts, then the ethical rationale for undertaking bias mitigation is obviously stronger. Bias impact is also a function of disease *prevalence*. If the clinical risk of model-driven inaccuracy is high for a given group, for instance due to the high prevalence of a condition in that patient class, then we have a stronger reason to mitigate bias in the model. The above quantitative aspects, albeit not being presented as fixed thresholds, integrate with qualitative considerations in ethical reasoning about the need to address disparities across different patient classes.

Once they have established that uneven performance across patient groups is significant, that it affects a substantial number of patients, and that it should be corrected, developers can select among available debiasing techniques. To orient such decisions from an ethical point of view,

=> **[A3.2] Developers should define a fairness goal, that is, decide what is the ethical objective of reducing disparity in model performance.**

Defining a fairness goal means stating what is the ethical criterion to follow when attempting to reduce observed disparities. Two well-established ethical criteria exist to this effect: the egalitarian and the maximin (i.e., *maxi*mize the *min*imum) principle.

The egalitarian principle suggests choosing the mitigation that minimizes the performance difference (delta) between the worst- and the best-off classes. Another way to interpret the egalitarian principle is to look at how model performance spreads around its median, that is, trying to reduce standard deviation.

The maximin principle, instead, requires choosing the mitigation that is most favorable to the worst-off class [26]. The maximin principle can also be interpreted as ensuring a minimum performance threshold that even the worst-off group is supposed to meet, independently of how the overall delta and performance spread are affected.

Specific circumstances can provide contextual guidance to select the most appropriate fairness goal. For instance, if the worse off group is large, or more at risk of serious clinical complications due to model miscalls (as evidenced for instance through the metric of equalized odds), a model that, following the maximin principle, boosts performance for this group is preferable over a model that reduces the gap between best and worst off but does not sufficiently improve performance for the more at-risk group. Developers should be able to document and justify (via clinical as well as ethical considerations) their choice of the most adequate fairness goal informing their bias mitigation strategy.

Model developers have a variety of methods to improve fairness during model development [19,26].

=> **[A3.3] Developers should adopt the most appropriate debiasing technique to meet their fairness goal.**

Debiasing techniques can be implemented at various stages in the ML pipeline [27]: at pre-processing stage, before data input to the model (for instance, by adjusting sample weights, modifying label definitions, or through artificial imputation of missing data points[28]); during model training (known as in-process, such as incorporating independence of the protected attribute in the loss function); or post processing after model predictions are generated, as in the case of score recalibration for specific sub-cohort [29]. The identified sources of bias can guide the choice of debiasing techniques, hinting at which aspects of the dataset or steps in the ML pipeline need to be fixed. However, since all debiasing techniques come with specific limitations, debiasing should be understood as a work of approximation.

Trade-offs between model fairness and model performance can be complex. How much performance deterioration is acceptable, and how much performance improvement is needed in light of a given fairness goal will depend on further ethical considerations.

We consider algorithmic fairness and accuracy *prima facie* of equal importance, or not lexicographically ordered [30]. This is why, when the requirements of fairness and those of accuracy conflict, their trade-off requires ethical consideration. Addressing ethical trade-offs amounts to what in practical ethics is referred to as balancing, that is, defining the strength or importance of one principle or norm with respect to the other in specific circumstances. To the extent that measurable deterioration of either fairness or performance affects patients, optimal balancing depends on qualitative considerations about patient well-being.

Different mitigation techniques can give rise to different losses in model performance [31]. A key ethical condition for the choice of strategies to improve model fairness is that bias mitigation does not result in a considerable loss of overall performance. But just how much performance deterioration can be acceptable is a highly context-dependent issue. Comparing how different mitigation strategies affect model performance in the aggregate, gives developers an indication to select the best performing mitigation strategy. Several studies have conducted quantitative analyses of the trade-offs between fairness and performance when applying different mitigation strategies [32]. These works provide practical insights into how different approaches can impact model performance across subgroups. As a general rule, performance deterioration should not be such as to precipitate the model below what can be considered the standard of care in a given field. When different bias mitigation techniques can be adopted, the one that degrades performance the least should be selected. That this is not, however, an absolute rule: an exception is represented by cases in which one class of patients is more at risk even in case of minor deterioration in model performance, e.g., due to age or pre-existing clinical conditions. In cases in which fairness cannot be mitigated without deteriorating the model below the standard of care, the model should not be used, or its use be restricted to classes of patients for which it works well.

There can be more than one alternative to meet the defined fairness goal, and bias mitigation can result in different degrees of performance deterioration for different classes of patients (Box 3). Such differences can be used to assess model performance deterioration in qualitative terms, that is, in light of context and task-specific characteristics such as the severity of the clinical risks related to the use of the model, or class-specific risks (Box 4, Box 5, Box 6).

**Box 3. Measuring model fairness**

Given the nature of the clinical task at hand, and the life-threatening risk of false negative outputs, we take equality of opportunity (TPR, aka recall or sensitivity) as the ethically more appropriate metric to measure model fairness across different clinical and demographic cohorts. We can thus measure equality of opportunity across the previously identified dimensions (gender, age, and clinical indication) using TPR as well as metrics that take TPR into account. These include the area under the receiving operator curve (AUROC) and the area under the precision-recall curve (AUPRC). The AURPC computes the trade-off between precision (PPV) and recall (TPR) in a given model and, contrary to AUROC - that computes fall-out (FPR) and recall (TPR)–is sensitive to disease prevalence.

**Fig 1:**
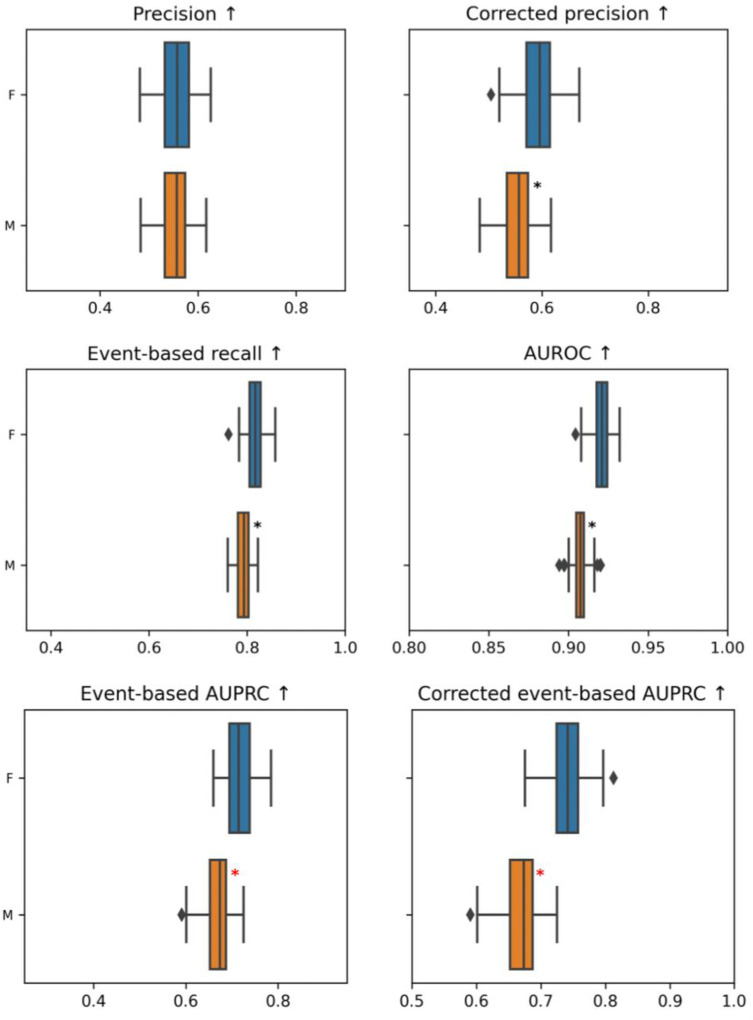
Comparison of different model outputs for male (M) and female (M) patients. The box plots illustrate the interquartile ranges (distance between 25th to 75th percentiles) computed by bootstrapping the patient population of the test set (drawing with replacement 100 random samples of the test set size, the line inside the box indicates the median, whiskers extend to 1.5 times the interquartile range, and outliers are plotted individually. Category cohorts in which metric is significantly worse off than the rest of the patients are indicated with a *black star*. According to each metric, 3 category cohorts (across all groupings: sex, age, clinical categories) with the largest deltas that are also significantly worse off than the rest of the population are flagged with a *red star*.

**Fig 2:**
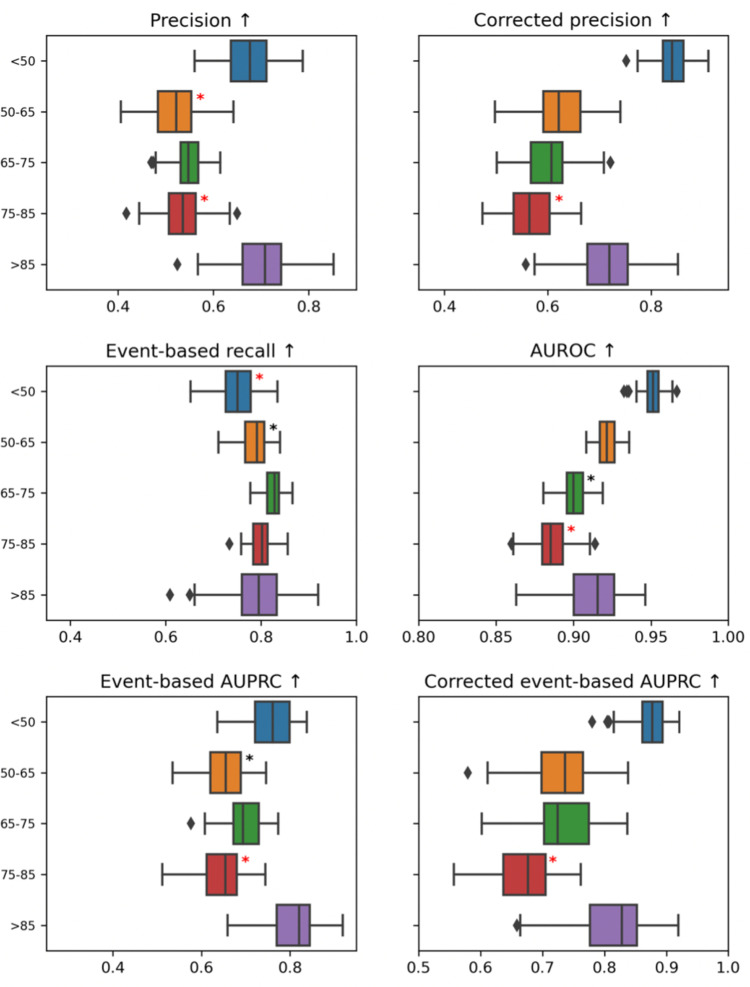
Comparison of model performance metrics across different age groups. The box plots illustrate the interquartile ranges (distance between 25th to 75th percentiles) computed by bootstrapping the patient population of the test set (drawing with replacement 100 random samples of the test set size, the line inside the box indicates the median, whiskers extend to 1.5 times the interquartile range, and outliers are plotted individually. Category cohorts in which metric is significantly worse off than the rest of the patients are indicated with a *black star*. According to each metric, 3 category cohorts (across all groupings: sex, age, clinical categories) with the largest deltas that are also significantly worse off than the rest of the population are flagged with a *red star*.

**Fig 3:**
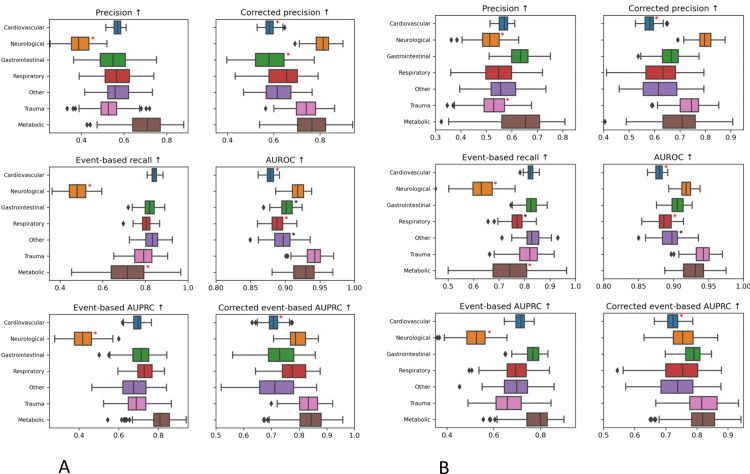
Comparison of different model performance metrics across different clinical categories: A) for the original model, B) after the label correction for neurological patients. The box plots illustrate the interquartile ranges (distance between 25th to 75th percentiles) computed by bootstrapping the patient population of the test set (drawing with replacement 100 random samples of the test set size), the line inside the box indicates the median, whiskers extend to 1.5 times the interquartile range, and outliers are plotted individually. Category cohorts in which metric is significantly worse off than the rest of the patients are indicated with a *black star*. According to each metric, 3 category cohorts (across all groupings: sex, age, clinical categories) with the largest deltas that are also significantly worse off than the rest of the population are flagged with a *red star*.

More specifically, we use *event-based* measures of TPR in a 12 hour timeframe (i.e., for each event we check whether an alarm has been triggered in the previous 12 hours), that is: event-based recall; event-based AUPRC; corrected event-based AUPRC (Figs 1–3). *Corrected* event-based AUPRC measures refer to normalized prevalence across the compared cohorts.

We have computed fairness metrics in each selected subgroup and compared median values using the following formula:

$$\Delta =\left|\text{median}{\left\{{\text{metric}}_{p\in S_{n}\cap G}\right\}}_{n=1}^{N}-\text{median}{\left\{{\text{metric}}_{p\in S_{n}\cap \bar{G}}\right\}}_{n=1}^{N}\right|,$$

where *p* is a patient or data point, $S_{n}$ is the *n* -th bootstrapped sample, *N* is the total number of bootstrapped samples, *G* is the studied patient class, $\bar{G}$ is the rest of the population.

We compare the distribution of each median from one cohort to the rest of the population using the Mann-Whitney U test with Bonferroni correction to account for multiple comparisons.

**Box 4. Gender fairness**

Upon checking the model via different statistical formalizations of fairness, we noticed deteriorated performance in male vs female patients (see Fig 1). Note that gender bias in the model is favorable to women. Gender differences are statistically significant, but the delta between these two groups is small, and it could be considered ethically acceptable. The disparity in event-based recall results in 23 additional undetected circulatory failures for male patients considering 1000 male patients versus 1000 female patients with the same number of failures.

**Box 5. Age fairness**

Our model’s performance in terms of event-based recall deteriorated for younger age groups (patients below 65 years old), while for other metrics such as precision, performance is reduced for patients aged 50 to 85 (see Fig. 2). We have noticed that reduced event-based recall is in principle acceptable for younger patients as they face lesser risks of major adverse outcomes due to model miscalls. Precision could be improved for older patients if it does not result in a noticeable reduction in event-based recall.

**Box 6. Clinical fairness**

Neurological patients are the worst-performing group in terms of event-based recall and event-based AUPRC. This deteriorated performance most likely reflects label bias as discussed above (see Box 1). This warrants altering the proxy definition, for instance by increasing the arterial pressure threshold for neurological patients by 10 mmHg.

Since the model behaves rather evenly for all other clinical groups, the fairness goal here should be to boost model performance for the obvious outlier: neurological patients (maximin principle).

The change in proxy definition results in an improvement in the event-based recall of neurological patients (going from 0.48 to 0.631; Fig 3). Note that, while this resulted in a smaller overall delta value for event-based recall (from 0.346 to 0.185), it increased scatter and dispersion, albeit only marginally, due to a small degradation in performance for respiratory patients.

We also tried some more common mitigation techniques such as recalibrating the output score per subcohort and training submodels, however they didn’t improve the performance for neurological patients. This shows the benefit of identifying sources of bias to construct a relevant and efficient debiasing technique; by spotting the origin of the problem we have been able to directly address it.

### 4. Outcome fairness: Measuring outcome fairness

During model implementation, specific sources of bias can lead to uneven patient outcomes. When a model is deployed in any given clinical setting, actual patients may have specific socio-demographic characteristics that do not necessarily match training and validation data (training-serving skew, Table 2), leading to worse health outcomes for patient groups that were not represented in the training data. This type of assessment is not always possible, depending on available information and metadata. In the absence of socio-demographic information in training and validation datasets, ad hoc monitoring should be in place at the site where the model is in use to compare health outcomes across different socio-demographic patient groups. ML training and validation datasets are representative if estimates obtained on that sample are generalizable to the target population [33], therefore, prior to model implementation

=> **[A4.1] Discrepancies between the clinical and socio-demographic composition of the actual patient population and the composition of the training and validation data should be documented.**

In other words, developers should check for the possibility that training-serving skew (see Table 2) may lead to worse health outcomes for patient groups that were not represented in the training data. If relevant discrepancies in clinical features or protected attributes are detected

=> **[A4.2] Regular monitoring should be set up to promptly detect signs of inferior outcomes for patient groups not sufficiently represented in the original data.**

Outcome-related disparities can depend on the model itself or on its users. Variations in health outcomes due to model predictions and assessments are **model-dependent**. While model dependent biases such as the time difference between alarm and event can be measured during model development (e.g., on a separate validation dataset), how such characteristic affects patient outcomes (model-dependent biases) can be measured ex post.

On the other hand, outcome fairness depends also on how a model works and is operated in real-world clinical conditions, for instance how users such as healthcare professionals (HCPs) respond to its output, or translate model output into clinical decisions. Differential outcomes resulting from operational conditions are **user-dependent** and can as well be detected once the model is implemented. It is possible, for instance, that a healthcare practitioner relies too much on the input of a ML model without questioning its predictions in light of her expertise and experience; or that one tends to–consciously or not–ignore input from a ML tool, for example by dismissing alerts that pop up too frequently in what is known as alarm fatigue [34]. Moreover, end-users have their own biases too. It is thus possible that automation and dismissal bias is more prevalent in the case of certain patient groups than others, with obvious consequences in terms of uneven health outcomes.

Only the nature of the clinical task at hand can suggest the risk of biased user-dependent outcomes (see Box 7). Therefore

=> **[A4.3] Developers should document the anticipated risk of user-dependent outcome biases.**

and

=> **[A4.4] Regular monitoring and statistical trend analysis should be in place to measure differences in how the model is operated for different clinical and socio-demographic patient groups.**

Data should be collected on how practitioners use a model - meaning how often they follow their advice and what courses of action result from its use - and compute use-patterns with patient characteristics and health outcomes, so as to monitor whether and to which extent the model produces fair health outcomes for all patient types. There is ample room to automate such monitoring to spot potential sources of outcome bias during clinical use across different socio-demographic and clinical classes, so as to enable prompt corrective actions in case uneven outcomes or deteriorating trends are detected.

**Box 7. Outcome fairness**

Given the absence of socio-demographic specifications in our original data, we could not control for minority bias. Moreover differences between training and validation data and the actual clinical population at the site of model implementation cannot be documented prior to model implementation. Ad hoc monitoring should be in place to compare health outcomes across different socio-demographic classes while the model is in use.

We reasoned that an excessive false positive rate (FPR) can create inefficiencies in model implementation linked to dismissal bias (alarm fatigue) on the part of healthcare professionals. FPR can be checked during model development but proper adjustments also depend on the implementation context and can thus only be made ex post. This risk has to be reported and documented upon model implementation so that adequate monitoring and personnel training can be set up.

Moreover, our model’s performance does not depend exclusively on a correct binary classification (whether or not the patient will experience circulatory failure), but also on the timing of the warning relative to the actual manifestation of circulatory failure. We can therefore check whether the model triggers alarms sufficiently in advance for different cohorts of patients by comparing time gaps between the first accurate alarm and circulatory failure. Such measurements should be reported and documented upon model implementation to allow clinical users to adapt their monitoring and intervention protocols.

The risk that automation bias affects health outcomes for our model is low because a wrong prediction like a false alarm–while undesirable in terms of clinical workflow–is unlikely to endanger patients’ health.

### 5. Improving outcome fairness

Patient outcomes should be recorded automatically so as to enable periodic monitoring of the model in terms of safety and effectiveness. Outcome bias can be due to the interplay of model-dependent, user-dependent and other context-specific conditions, making its mitigation a particularly complex and uncertain undertaking. Model-dependent differences in health outcomes, for instance, can be addressed by considering the role of protected attributes bearing in mind that predictive clinical algorithms taking race or ethnicity into account, for instance, can lead to worse health outcomes for minorities [35]. Nevertheless, suspected sources of outcome bias should be identified, monitored and, if needed, corrected.

In the presence of worse outcomes for specific patient classes,

=> **[A5.1] Models should be fine-tuned or customized (including with ad hoc re-training on new data) to match the characteristics of the patient cohort in which they are being used** [36**].**

Restricting model use to groups for which outcomes are acceptable is also an option. Other forms of model optimization can address user-dependent biases. Identified risks should be documented to enable end-user training. If, despite training, automation or dismissal bias is detected, users should be alerted and where appropriate re-trained about optimal model use.

=> **[A5.2] End-users should be adequately instructed on optimal model use–which includes correcting biased practices via targeted training sessions.**

## Discussion

While bias in statistical models is not specific to ML, ML fairness has so far received considerable attention, including in the space of medical AI [37–40]. Many have stressed that available health data are not sufficiently inclusive of patients with different socio-economic characteristics. In fact, for a disconcerting percentage of ML models, training datasets do not even report variables such as ethnicity, gender and age [41]. Moreover, the root causes of outcome bias can be analyzed, but the opaque nature of ML algorithms constitutes an obstacle to the full understanding of a model’s behavior, especially in relation to individual patient decisions [42]. ML systems are black-boxes in the sense that it is not possible to gain access to the myriad rules those models have learnt to associate input and output. Such opacity stands in the way of capturing what processes in a given model lead to biased outputs or outcomes. Interpretable models can offer some closer insights, by for instance shedding light on which features in the data have more weight in model predictions. But AI cannot be made interpretable by design–so as to be sure that it behaves fairly for all classes of input data: in fact, so called explainable AI, amounts to an opaque AI systems analyzing other opaque AI systems. Therefore while explainable AI could help in the analysis of biased algorithms, it does not replace the need to test and monitor how a model treats different patients’ classes.

In light of such considerations, it is crucial to orient technical choices in ethically robust ways that take into due account the contextual specificities of the clinical task at hand, as well as the conditions that determine model impact on individual health.

**Fig 4:**
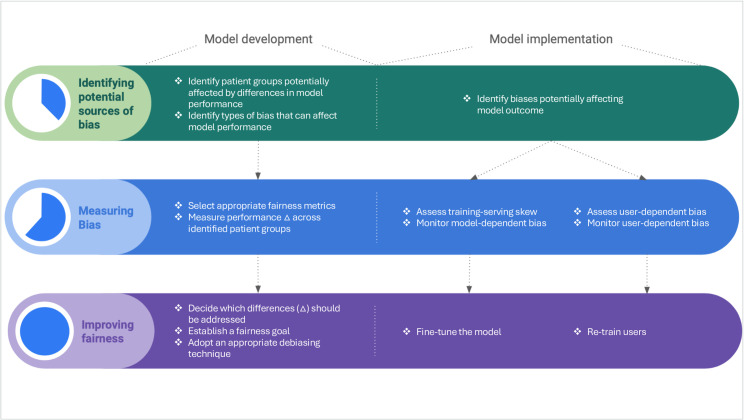
A fairness framework for medical ML.

Numerous frameworks have been introduced to help developers cope with bias in ML across a model’s entire life-cycle [43–46]. Here we have endorsed such a life-cycle approach (Fig 4) and introduced action points and technical requirements to adequately operationalize our proposed criteria in practice from model development to clinical implementation (Table 3). Our framework seamlessly integrates ethical and technical requirements ensuring that bias mitigation choices can be appropriately justified and documented, also facilitating oversight mechanisms such as ethics-based auditing of AI systems [47].

**Table 3:** Summary of the action points for bias identification, measurement, and mitigation.

Domains	#	Actions
*Bias identification*	**A1**	Developers should identify potential sources of bias to define for which classes of patients the model may under-perform.
*Measuring model fairness*	**A2.1**	Developers should define which mathematical formalization of fairness is best suited to the clinical task at hand.
	**A2.2**	Developers should comparatively assess model performance across the identified patients classes based on the fairness metric(s) they have selected.
*Improving model fairness*	**A3.1**	Developers should decide which of the identified disparities across patient groups should be corrected.
	**A3.2**	Developers should define a fairness goal, that is, decide what is the ethical objective of reducing disparity in model performance.
	**A3.3**	Developers should adopt the most appropriate debiasing technique to meet their fairness goal.
*Measuring outcome fairness*	**A4.1**	Discrepancies between the clinical and socio-demographic composition of the actual patient population and the composition of the training and validation data should be documented.
	**A4.2**	Regular monitoring should be set up to promptly detect signs of inferior outcomes for patient groups not sufficiently represented in the original data.
	**A4.3**	Developers should document the anticipated risk of user-dependent outcome biases.
	**A4.4**	Regular monitoring and statistical trend analysis should be in place to measure differences in how the model is operated for different clinical and socio-demographic patient groups.
*Improving outcome fairness*	**A5.1**	Models should be fine-tuned or customized (including with ad hoc re-training on new data) to match the characteristics of the patient cohort in which they are being used.
	**A5.2**	End-users should be adequately instructed on optimal model use–which includes correcting biased practices via targeted training sessions.

To a great extent, the manifestation and the impact of algorithmic bias is a highly context-dependent phenomenon. Nonetheless, issues revolving around the magnitude and the impact of model bias are key to determine the ethically most appropriate solutions.

In terms of generalizability, we are confident that our framework can be applied to a wide variety of clinical specialties and use cases. In fact, none of our action-points is specific to a particular field of medicine, nor the framework is based on a closed set of patient characteristics–even if some of the attributes along which bias typically emerges are recurrent in medicine [48]. The application of our framework in the context of different use cases will require further specification of its action-guides, that is, narrowing down their scope in order to capture how bias is produced across different relevant socio-demographic and clinical characteristics.

A special case is represented by federated learning ecosystems. In such scenarios, fairness should be measured and ensured locally before clinical implementation. While comparing demographics for both training and input data across the federated ecosystem may be more problematic, it is still possible in pseudonymized or anonymized format. Moreover, in federated ecosystems data quality can be improved by standardizing data collection across different nodes, which will also reduce the likelihood of bias due for instance to data missingness.

More in general, data quality issues should be addressed across all the known dimensions of the problem, including data completeness (whether data contain all relevant information about patients), correctness (whether data reflect true information about patients), concordance (whether information about patients is consistent across different datasets), plausibility (whether data make sense in light of other data or information about a patient), currency (whether data are up to date), and accessibility (whether data are equally accessible) [49].

Emerging legislation can help address data quality as well as other pressing ethical issues in clinical AI [50]. Our framework is neutral with respect to specific regulatory conditions, but we do not anticipate that it would conflict with commonly adopted rules around the use of medical software or digital health technologies. Quite to the contrary, our framework offers the opportunity to comply with emerging regulations. For instance, in Europe, the AI Act [51] offers comprehensive provisions to address some well-known issues in AI, including clinical AI, but only contains generic provisions about fairness with respect to the quality of training datasets (art. 10 (f)). In this respect, our model is not only compatible with provisions about the detection, prevention and mitigation of biases (art. 10 (g)), but it also offers a framework to comply with such provisions. At any rate, hard law provisions are unlikely to include effective fairness requirements given the complex nature of ML bias and the inevitable trade-offs that arise when developers try to mitigate bias. Each model has its own specific biases, and how such biases translate in terms of health outcomes is hard to know–let alone to mitigate–a priori. Our framework shows that considerable effort is needed on the part of both developers and users to document bias, to monitor model behavior including in real-world conditions, and to tailor each model to the actual conditions of its use. To this aim, the importance of stakeholders’ involvement as a way to foster responsible innovation should not be overlooked [52,53]^,^. Meaningfully involving different stakeholders in improving model fairness is not straightforward. Yet, best practices of co-creation and regulatory sandboxing are emerging as plausible options to allow fast-paced fields such as medical AI to progressively move in this direction [53].

## Conclusion

The integration of ML in medicine holds immense promise for enhancing healthcare, yet it brings forth intricate ethical challenges, particularly regarding algorithmic bias and model fairness.

Our work presents a blueprint to enable proportionate bias mitigation, addressing inevitable trade-offs, and improving accountability for both developers and clinical. By bridging the gap between technical and ethical requirements, this proposed framework strives to cultivate fairness, transparency and enhanced healthcare outcomes in the realm of clinical ML. As the clinical AI landscape evolves, embracing a practical ethics framework within ML applications becomes paramount to enable responsible innovation, and effective oversight.

## References

[1] Richardson HS. Specifying norms as a way to resolve concrete ethical problems. Philos. Public Aff. 1990;19(4):279–310.

[2] Wailoo KA, Dzau VJ, Yamamoto KR. Embed equity throughout innovation. Science. 2023;381(6662):1029. doi: 10.1126/science.adk6365 37676960

[3] Pot M, Kieusseyan N, Prainsack B. Not all biases are bad: equitable and inequitable biases in machine learning and radiology. Insights Imaging. 2021;12(1):13. doi: 10.1186/s13244-020-00955-7 33564955 PMC7872878

[4] Grote T, Keeling G. Enabling fairness in healthcare through machine learning. Ethics Inf Technol. 2022;24(3):39. doi: 10.1007/s10676-022-09658-7 36060496 PMC9428374

[5] McCradden MD, Joshi S, Mazwi M, Anderson JA. Ethical limitations of algorithmic fairness solutions in health care machine learning. Lancet Digit Health. 2020;2(5):e221–3. doi: 10.1016/S2589-7500(20)30065-0 33328054

[6] Corbett-Davies S, Gaebler J, Nilforoshan H, Shroff R, Goel S. The measure and mismeasure of fairness. J. Mach. Learn. Res. 2023;24(1):14730–846.

[7] Powers M, Faden R. Structural injustice: power, advantage, and human rights. Oxford University Press; 2019.

[8] Powers M, Faden RR. Social justice: the moral foundations of public health and health policy. Oxford University Press; 2006. p. 258.

[9] Daniels N. Just health: meeting health needs fairly. Cambridge University Press; 2008.

[10] Degrazia D, Beauchamp T. Philosophy: ethical principles and common morality. Methods Med. Ethics 2010;2010:21–34.

[11] Beauchamp TL. Principlism and its alleged competitors. Kennedy Inst Ethics J. 1995;5(3):181–98. doi: 10.1353/ken.0.0111 11645305

[12] Richardson HS. Specifying, balancing, and interpreting bioethical principles. J Med Philos. 2000;25(3):285–307. doi: 10.1076/0360-5310(200006)25:3;1-H;FT285 10852336

[13] Beauchamp TL. Methods and principles in biomedical ethics. J Med Ethics. 2003;29(5):269–74. doi: 10.1136/jme.29.5.269 14519835 PMC1733784

[14] Yèche H, Kuznetsova R, Zimmermann M, Hüser M, Lyu X, Faltys M, et al. HiRID-ICU-Benchmark -- A Comprehensive Machine Learning Benchmark on High-resolution ICU Data. 2021; Available from: https://arxiv.org/abs/2111.08536.

[15] Faltys M, Zimmermann M, Lyu X, Hüser M, Hyland S, Rätsch G, et al. HiRID, a high time-resolution ICU dataset (version 1.1. 1). Physio Net. 2021;10.

[16] Danks D, London AJ. Algorithmic Bias in Autonomous Systems. In 2017. p. 4691–7.

[17] Celi LA, Cellini J, Charpignon M-L, Dee EC, Dernoncourt F, Eber R, et al. Sources of bias in artificial intelligence that perpetuate healthcare disparities-A global review. PLOS Digit Health. 2022;1(3):e0000022. doi: 10.1371/journal.pdig.0000022 36812532 PMC9931338

[18] Rajkomar A, Hardt M, Howell MD, Corrado G, Chin MH. Ensuring fairness in machine learning to advance health equity. Ann Intern Med. 2018;169(12):866–72. doi: 10.7326/M18-1990 30508424 PMC6594166

[19] Pagano TP, Loureiro RB, Lisboa FVN, Peixoto RM, Guimarães GAS, Cruz GOR, et al. Bias and unfairness in machine learning models: a systematic review on datasets, tools, fairness metrics, and identification and mitigation methods. BDCC. 2023;7(1):15. doi: 10.3390/bdcc7010015

[20] Beigang F. Yet another impossibility theorem in algorithmic fairness. Minds Mach. 2013:1–21.

[21] Saravanakumar KK. The impossibility theorem of machine fairness -- a causal perspective [Internet]. arXiv. 2021. Available from: http://arxiv.org/abs/2007.06024

[22] Kleinberg J, Mullainathan S, Raghavan M. Inherent trade-offs in the fair determination of risk scores [Internet]. arXiv. 2016. Available from: http://arxiv.org/abs/1609.05807.

[23] Friedler SA, Scheidegger C, Venkatasubramanian S. On the (im)possibility of fairness [Internet]. arXiv. 2016. Available from: http://arxiv.org/abs/1609.07236.

[24] Chen IY, Pierson E, Rose S, Joshi S, Ferryman K, Ghassemi M. Ethical machine learning in healthcare. Annu Rev Biomed Data Sci. 2021;4:123–44. doi: 10.1146/annurev-biodatasci-092820-114757 34396058 PMC8362902

[25] Hoche M, Mineeva O, Burger M, Blasimme A, Ratsch G. FAMEWS: a fairness auditing tool for medical early-warning systems. In: Pollard T, Choi E, Singhal P, Hughes M, Sizikova E, Mortazavi B, et al., editors. Proceedings of the fifth conference on health, inference, and learning [Internet] (vol. 248). PMLR; 2024. p. 297–311. Available from: https://proceedings.mlr.press/v248/hoche24a.html

[26] Rawls J. A Theory of Justice: Original Edition. Harvard University Press; 1971 [cited 2024 Jul 16]. Available from: https://www.jstor.org/stable/j.ctvjf9z6v

[27] Pessach D, Shmueli E. A review on fairness in machine learning. ACM Comput Surv. 2022;55(3):1–44. doi: 10.1145/3494672

[28] Jeanselme V, De-Arteaga M, Zhang Z, Barrett J, Tom B. Imputation strategies under clinical presence: Impact on algorithmic fairness. In PMLR; 2022. p. 12–34. PMC7614014 36601036

[29] Salvador T, Cairns S, Voleti V, Marshall N, Oberman A. FairCal: fairness calibration for face verification. arXiv. 2022. Available from: http://arxiv.org/abs/2106.03761

[30] Childress JF. The place of autonomy in bioethics. Hastings Center Report. 1990;20(1):12–7. 2179164

[31] Feldman M, Friedler S, Moeller J, Scheidegger C, Venkatasubramanian S. Certifying and removing disparate impact. arXiv. 2015;abs/1412.3756.

[32] Friedler SA, Scheidegger C, Venkatasubramanian S, Choudhary S, Hamilton EP, Roth D. A comparative study of fairness-enhancing interventions in machine learning. In: Proceedings of the conference on fairness, accountability, and transparency [Internet]. New York, NY, USA: Association for Computing Machinery; 2019. p. 329–38. (Fat*’19). Available from: doi: 10.1145/3287560.3287589

[33] Rudolph JE, Zhong Y, Duggal P, Mehta SH, Lau B. Defining representativeness of study samples in medical and population health research. BMJ Med. 2023;2(1):e000399. doi: 10.1136/bmjmed-2022-000399 37215072 PMC10193086

[34] Mitka M. Joint commission warns of alarm fatigue: multitude of alarms from monitoring devices problematic. JAMA. 2013;309(22):2315–6. doi: 10.1001/jama.2013.6032 23757063

[35] Vyas DA, Eisenstein LG, Jones DS. Hidden in plain sight - reconsidering the use of race correction in clinical algorithms. N Engl J Med. 2020;383(9):874–82. doi: 10.1056/NEJMms2004740 32853499

[36] Aristidou A, Jena R, Topol E. Bridging the chasm between AI and clinical implementation. Lancet. 2022;399(10325):620. 35151388 10.1016/S0140-6736(22)00235-5

[37] Chen IY, Joshi S, Ghassemi M. Treating health disparities with artificial intelligence. Nat Med. 2020;26(1):16–7. doi: 10.1038/s41591-019-0649-2 31932779

[38] Varoquaux G, Cheplygina V. Machine learning for medical imaging: methodological failures and recommendations for the future. NPJ Digit Med. 2022;5(1):48. doi: 10.1038/s41746-022-00592-y 35413988 PMC9005663

[39] Teno JM. Garbage in, garbage out—words of caution on big data and machine learning in medical practice. In American Medical Association; 2023. p. e230397–e230397. 10.1001/jamahealthforum.2023.0397 36795395

[40] Goldberg CB, Adams L, Blumenthal D, Brennan PF, Brown N, Butte AJ, et al. To do no harm — and the most good — with ai in health care. NEJM AI. 2024;1(3). doi: 10.1056/aip2400036 38388841

[41] Bozkurt S, Cahan EM, Seneviratne MG, Sun R, Lossio-Ventura JA, Ioannidis JPA, et al. Reporting of demographic data and representativeness in machine learning models using electronic health records. J Am Med Inform Assoc. 2020;27(12):1878–84. doi: 10.1093/jamia/ocaa164 32935131 PMC7727384

[42] Ghassemi M, Oakden-Rayner L, Beam AL. The false hope of current approaches to explainable artificial intelligence in health care. Lancet Digit Health. 2021;3(11):e745–50. doi: 10.1016/S2589-7500(21)00208-9 34711379

[43] Hwang TJ, Kesselheim AS, Vokinger KN. Lifecycle regulation of artificial intelligence- and machine learning-based software devices in medicine. JAMA. 2019;322(23):2285–6. doi: 10.1001/jama.2019.16842 31755907

[44] Vokinger KN, Feuerriegel S, Kesselheim AS. Mitigating bias in machine learning for medicine. Commun Med (Lond). 2021;1:25. doi: 10.1038/s43856-021-00028-w 34522916 PMC7611652

[45] McCradden MD, Anderson JA, A Stephenson E, Drysdale E, Erdman L, Goldenberg A, et al. A research ethics framework for the clinical translation of healthcare machine learning. Am J Bioeth. 2022;22(5):8–22. doi: 10.1080/15265161.2021.2013977 35048782

[46] Price WN II, Sendak M, Balu S, Singh K. Enabling collaborative governance of medical AI. Nat Mach Intell. 2023;5(8):821–3. doi: 10.1038/s42256-023-00699-1

[47] Mökander J, Floridi L. Ethics-based auditing to develop trustworthy AI. Minds & Machines. 2021;31(2):323–7. doi: 10.1007/s11023-021-09557-8

[48] FitzGerald C, Hurst S. Implicit bias in healthcare professionals: a systematic review. BMC Med Ethics. 2017;18(1):19. doi: 10.1186/s12910-017-0179-8 28249596 PMC5333436

[49] Weiskopf NG, Weng C. Methods and dimensions of electronic health record data quality assessment: enabling reuse for clinical research. J Am Med Inform Assoc. 2013;20(1):144–51. doi: 10.1136/amiajnl-2011-000681 22733976 PMC3555312

[50] Prainsack B, Forgó N. New AI regulation in the EU seeks to reduce risk without assessing public benefit. Nature Medicine. 2024:1–3. 10.1038/s41591-024-02874-2 38499661

[51] Regulation - EU - 2024/1689 - EN - EUR-Lex.

[52] Landers C, Vayena E, Amann J, Blasimme A. Stuck in translation: Stakeholder perspectives on impediments to responsible digital health. Front Digit Health. 2023;5:1069410. doi: 10.3389/fdgth.2023.1069410 36815171 PMC9939685

[53] Landers C, Blasimme A, Vayena E. Sync fast and solve things-best practices for responsible digital health. NPJ Digit Med. 2024;7(1):113. doi: 10.1038/s41746-024-01105-9 38704413 PMC11069566

